# Induction and regulation of matrix metalloproteinase-12in human airway smooth muscle cells

**DOI:** 10.1186/1465-9921-6-148

**Published:** 2005-12-16

**Authors:** Shaoping Xie, Razao Issa, Maria B Sukkar, Ute Oltmanns, Pankaj K Bhavsar, Alberto Papi, Gaetano Caramori, Ian Adcock, K Fan Chung

**Affiliations:** 1 Experimental Studies, National Heart and Lung Institute, Imperial College, London, UK; 2 Centro di Ricerca su Asma e BPCO, University of Ferrara, Ferrara, Italy

## Abstract

**Background:**

The elastolytic enzyme matrix metalloproteinase (MMP)-12 has been implicated in the development of airway inflammation and remodeling. We investigated whether human airway smooth muscle cells could express and secrete MMP-12, thereby participating in the pathogenesis of airway inflammatory diseases.

**Methods:**

Laser capture microdissection was used to collect smooth muscle cells from human bronchial biopsy sections. MMP-12 mRNA expression was analysed by quantitative real-time RT-PCR. MMP-12 protein expression and secretion from cultured primary airway smooth muscle cells was further analysed by Western blot. MMP-12 protein localization in bronchial tissue sections was detected by immunohistochemistry. MMP-12 activity was determined by zymography. The TransAM AP-1 family kit was used to measure c-Jun activation and nuclear binding. Analysis of variance was used to determine statistical significance.

**Results:**

We provide evidence that MMP-12 mRNA and protein are expressed by *in-situ *human airway smooth muscle cells obtained from bronchial biopsies of normal volunteers, and of patients with asthma, COPD and chronic cough. The pro-inflammatory cytokine, interleukin (IL)-1β, induced a >100-fold increase in MMP-12 gene expression and a >10-fold enhancement in MMP-12 activity of primary airway smooth muscle cell cultures. Selective inhibitors of extracellular signal-regulated kinase, c-Jun N-terminal kinase and phosphatidylinositol 3-kinase reduced the activity of IL-1β on MMP-12, indicating a role for these kinases in IL-1β-induced induction and release of MMP-12. IL-1β-induced MMP-12 activity and gene expression was down-regulated by the corticosteroid dexamethasone but up-regulated by the inflammatory cytokine tumour necrosis factor (TNF)-α through enhancing activator protein-1 activation by IL-1β. Transforming growth factor-β had no significant effect on MMP-12 induction.

**Conclusion:**

Our findings indicate that human airway smooth muscle cells express and secrete MMP-12 that is up-regulated by IL-1β and TNF-α. Bronchial smooth muscle cells may be an important source of elastolytic activity, thereby participating in remodeling in airway diseases such as COPD and chronic asthma.

## Background

Matrix metalloproteinases (MMPs) are a group of zinc-dependent structurally-related extracellular matrix (ECM) degrading proteinases that regulate ECM composition and are also able to cleave non-matrix proteins including growth factors, chemoattractants and cell surface receptors [1,2] There are more than 20 MMPs that can degrade every component of ECM and each MMP has its own substrate specificity [3-5]. Because of their ability to degrade ECM proteins, MMPs mediate tissue remodeling under physiological and pathological circumstances. The proteolytic activity of MMPs is counterbalanced by the presence of tissue inhibitors of metalloproteinases (TIMPs), which naturally inhibit MMPs by direct binding [6]. MMP-12, also called macrophage metalloelastase, was originally detected in alveolar macrophages of cigarette smokers [7]. It is secreted as a 54 kDa inactive pro-enzyme which is activated by proteolytic cleavage of the prodomain followed by processing into two active enzymes of 45 kDa and 22 kDa [7]. MMP-12 degrades a broad range of ECM proteins, including elastin, type IV collagen, fibronectin, laminin and gelatin [8,9], and is involved in turnover of the matrix, cell migration, tissue repairing and remodeling. In addition, MMP-12 can activate other MMPs, for example, MMP-2 and -3, leading to subsequent degradation of other ECM proteins [10].

MMP-12 may facilitate airway inflammation by stimulating migration of inflammatory cells such as monocytes and macrophages to inflammatory sites, and mediate airway remodeling by degrading ECM proteins through its enzymatic activity or through mediating inflammatory cytokines to induce other MMPs, including MMP-2, -9, -13 and -14, in lung [11]. Overproduction of MMP-12 causes pathological ECM protein breakdown and excessive airway remodeling, which has been implicated in a range of respiratory diseases, including asthma and chronic obstructive pulmonary disease (COPD). Studies from MMP-12 knock-out mice indicate that MMP-12 is a key mediator in cigarette smoke-induced emphysema [12].

Human airway smooth muscle cells (ASMC) express MMP-1, -2, -3, -9 and -14 [13-16]. The induction of MMP-12 by ASMC is however unknown. Considering the potential of ASMC to produce a host of soluble inflammatory mediators in response to inflammatory stimulation and their involvement in airway remodeling, we investigated the possibility that ASMC produce MMP-12. Since inflammatory cytokines have been shown to stimulate or inhibit MMP-12 induction in macrophages [17,18] and chondrocytes [19]), we examined the possible effects of the inflammatory cytokines, including interleukin (IL)-1β, tumour necrosis factor (TNF)-α and transforming growth factor (TGF)-β1, on MMP-12 induction of ASMC. Furthermore, we investigated the intracellular mechanisms of MMP-12 induction in ASMC, particularly the role of mitogen-activated protein kinases (MAPK), such as extracellular signal-regulated kinase (ERK) and c-Jun N-terminal kinase (JNK), and phosphatidylinositol 3-kinase (PI3-K) pathways.

## Methods

### Materials

All recombinant human cytokines were purchased from R&D Systems (Abingdon, UK). PD98059, SB203580, Wortmannin and LY294002 were obtained from Calbiochem (Nottingham, UK). SP600125 was a kind gift from Celgene (San Diego, CA). Primers for MMP-12 and GAPDH were purchased from Sigma Genosys (Pampisford, UK). Internal control 18S rRNA primers were provided by Applied Biosystems (Forster City, CA). Rabbit anti-human MMP-12 antibodies (AB19053 and AB19051) were obtained from Chemicon (Hampshire, UK). Precast gels and buffers for Western blot and zymography were purchased from Invitrogen (Paisley, UK). Nuclear extract kit and TransAM AP-1 family kit were from Active Motif (Rixensart, Belgium). RNase-free slides, reagents and other materials for Laser capture microdissection (LCM) were purchased from Arcturus (Hertfordshire, UK). Dexamethasone and all other tissue culture reagents were obtained from Sigma (Dorset, UK).

Human airway biopsies were obtained from normal volunteers (n = 4) and patients using the well-established procedures of fiberoptic bronchoscopy, and protocols that have been approved by the local Ethics Committee. The patients included three with moderate asthma, three with COPD and five with persistent 'idiopathic cough'. All subjects gave informed consent.

### Cell culture and treatment

Primary ASMC were isolated from fresh lobar or main bronchi, obtained from lung resection donors, by treatment with collagenase and cultured in DMEM supplemented with 10% FCS as described previously [20]. ASMC characteristics were identified by light microscopy with typical 'hill and valley' appearance and by positive immunostaining of smooth muscle (SM) α-actin, SM myosin heavy chain, calponin and SM-22. The cells were maintained in T175 culture flasks at 37°C in a humidified atmosphere of 5% CO_2_. For these experiments, ASMC were studied from passages 3–6.

Cells were trypsinized and subcultured in 6-well plates for total protein and RNA extractions or in T75 flasks for nuclear protein extraction. After reaching confluence in 10% FCS DMEM, cells were incubated for 2–3 days in serum-free medium containing 0.5% BSA before treatment. Cells were treated with IL-1β or the appropriate test reagents in fresh serum-free medium containing 0.5% BSA. Control cultures were incubated in the medium containing vehicle alone.

### Laser capture microdissection

Human airway biopsies were embedded in Optimum Cutting Temperature (OCT) compound on dry ice and snap-frozen in liquid nitrogen before storage at -80°C. Frozen sections were cut at 6 μm thickness and mounted on LCM slides (Arcturus). The slides were immediately stored on dry ice and then at -80°C until used. Sections were fixed in 70% ethanol for 30 seconds, and stained and dehydrated in a series of graded ethanol followed by xylene using HistoGene LCM frozen section staining kit (Arcturus) according to the manufacturer's instruction. ASMC were captured onto the CapSure HS LCM caps (Arcturus) by a Pixcell II Laser Capture Microdissection System (Arcturus, Mountain View, CA) and total RNA was extracted by using a PicoPure RNA isolation kit (Arcturus) according to the manufacturer's instructions.

### RT-PCR and real-time PCR

Total RNA was extracted from cultured ASMC by using the RNeasy Mini Kit (Qiagen, West Sussex, UK) according to the manufacturer's instructions. An aliquot of 0.5 μg total RNA was reverse transcribed using random hexamers and AMV reverse transcriptase (Promega). cDNA generated from 42 ng of total RNA was amplified by polymerase chain reaction (PCR) (RoboCycler, Stratagene, USA) or quantitative real-time PCR (Rotor Gene 3000, Corbett Research, Australia) using SYBR Green PCR Master Mix Reagent (Qiagen). The human MMP-12 forward and reverse primers were 5'-TGCTGATGACATACGTGGCA-3' and 5'-AGGATTTGGCAAGCGTTGG-3' 19). Each primer was used at a concentration of 2 μM or 0.5 μM for PCR or real-time PCR in each reaction. Cycling conditions for PCR were as follows: 95°C for 30 seconds; 60°C for 30 seconds followed by 72°C for 30 seconds for 30 cycles. The amplification products were analysed by 3% agarose gel electrophoresis. Cycling conditions for real-time PCR were as follows: step 1, 15 min at 95°C; step 2, 20 sec at 94°C; step3, 20 sec at 60°C; step 4, 20 sec at 72°C, with repeat from step 2 to step 4 for 35 times. Data from the reaction were collected and analysed by the complementary computer software (Corbett Research, Australia). Relative quantitations of gene expression were calculated using standard curves and normalized to GAPDH in each sample. For real-time PCR analysis of samples obtained from LCM, 18S rRNA was used as a housekeeping gene for internal control, and human lung tissue was used as a positive control.

### Western blotting

Total cell protein was extracted on ice with lysis buffer (1% Igepal CA-630, 0.5% sodium deoxycholate, 0.1% SDS in PBS pH 7.4) in the presence of freshly added protease inhibitors including 1 mM phenylmethylsulphonyl fluoride (PMSF), 5 μg/ml aprotinin, 1 mM Na_3_VO_4 _and 5 μg/ml leupeptin. Protein concentration was determined using the Bradford method with a Bio-Rad protein assay reagent. Protein extract (20 μg/lane) was fractionated by SDS-PAGE on a 10% tris-glycine precast gel and then transferred to a nitrocellulose membrane (Amersham). The membrane was incubated overnight at 4°C with an MMP-12 C-terminus antibody (0.5 μg/ml, AB19053) and then with an HRP-conjugated secondary antibody raised against rabbit IgG (1:2000, 1 hour) at room temperature. Antibody-bound proteins were visualised by ECL. The membranes were stripped and then reprobed with a mouse anti-GAPDH monoclonal antibody (1:5000, Biogenesis, Poole, UK) to control for protein loading. Relevant band intensities were quantified by scanning densitometric analysis using software from Ultra-Violet Products (Cambridge, UK). Densitometric data were normalized for GAPDH values.

To analyse the secretion of MMP-12, conditioned medium (400 μl) was concentrated to 20 μl by Centricon-10 miniconcentrator (Amicon, Bedford, MA) and fractionated by the 10% precast gel. Western blot analysis was performed as described above. Relevant band intensities were quantified by scanning densitometric analysis and normalised against the cell number (see below).

### Zymography

Conditioned media were harvested from ASMC cultures after treatments and the cell number in each well was detected by crystal violet assay [21]. MMP-12 activity was determined by gelatin zymography [22] according to the manufacturer's instructions (Invitrogen). Conditioned medium (20 μl) was fractionated by SDS-PAGE on a 10% precast zymography gel. To renature separated protein, gels were incubated 2 × 15 min with Renaturing Buffer (2.5% Triton-X 100; Invitrogen) by shaking, and then incubated with Developing Buffer (0.2% Brij; Invitrogen) for 30 min, followed by 18 hours incubation with Developing Buffer at room temperature by gentle shaking. Gels were stained in 0.1% Coomassie Brilliant Blue R-250 for approximately 1 hour and destained until the gelatinolytic bands were clearly seen. Gelatinolytic bands at 45kDa represent the active form of MMP-12. Relevant band intensities were quantified by scanning densitometric analysis and normalised to cell number.

### Immunohistochemistry and immunocytochemistry

Immunostaining was performed to detect the protein expression of MMP-12 in ASMC from human bronchial tissue sections or cell cultures on chamber slides. For Immunohistochemistry, bronchial samples were fixed in 4% formaldehyde and embedded in paraffin wax. 4 μm sections were cut before deparaffinization and rehydration. For immunocytochemistry, ASMC on chamber slides were fixed in 4% paraformaldehyde in PBS for 10 min followed by ice-cold acetone-methanol (50:50) for 10 min. Slides with both bronchial tissue section and ASMC layer were incubated in 3% hydrogen peroxide to block endogenous peroxidase activity, followed by 5% normal goat serum to block non-specific binding. Sections were incubated for 1 hour at room temperature with a rabbit anti-human MMP-12 hinge-region antibody (3.3 μg/ml, AB19051). Control slides were performed with normal rabbit immunoglobulin. Anti-rabbit biotinylated secondary antibody (Vector ABC Kit, Vector Laboratories) was applied to the sections for 30 min at room temperature, followed by the avidin/biotinylated peroxidase complex for another 30 min at room temperature. Sections were incubated with chromogenic substrate diaminobenzidine (DAB) for 5 min, and then counterstained in haematoxylin and mounted on aqueous mounting medium.

### c-Jun activation assay

Nuclear protein extracts were obtained from ASMC cultures by using the Nuclear Extract Kit (Active Motif) according to the manufacturer's instruction. Aliquots of nuclear protein were stored at -80°C. The activation of c-Jun was measured using the TransAM™ AP-1 family kit (Active Motif) according to the manufacturer's instruction. This method measures the DNA-binding activity of activator protein (AP)-1 by ELISA. Briefly, 5 μg of nuclear protein samples were incubated for 1 hour in a 96-well plate coated with an oligonucleotide that contains a TRE (5'-TGAGTCA-3'), to which phosphorylated c-Jun (p-c-Jun) contained in nuclear extracts specifically binds. After washing, p-c-Jun antibody (1:500 dilutions) was added to these wells and incubated for 1 hour. Following incubation for 1 hour with a secondary HRP-conjugated antibody (1:1000 dilution), specific binding was detected by colorimetric estimation at 450 nm with a reference wavelength of 655 nm.

### Data analysis

Data were analysed by analysis of variance (ANOVA) using the software program, Statview (Abacus Concept, Inc., Berkeley, CA, USA). Results are expressed as mean ± SEM and are representative of at least three separate experiments. P < 0.05 was used to determine the statistical significance.

## Results

### Expression of MMP-12 mRNA and protein by in-situ ASMC

**Figure 1:**
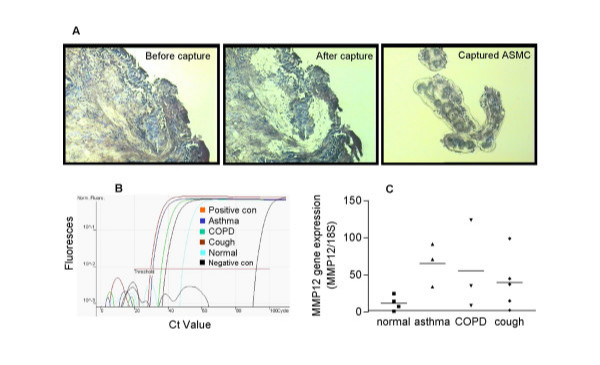
Expression of MMP-12 mRNA by *in-situ *ASMC. Laser capture microdissection was performed to collect ASMC from bronchial biopsy sections (A). MMP-12 mRNA expression was analysed by real-time RT-PCR (B). The data, obtained from 4 normal volunteers, 3 asthma, 3 COPD and 5 chronic cough patients, were normalized to the housekeeping gene 18S rRNA representative of relative MMP-12 mRNA expression (C). Lung tissue was used as a positive control.

To determine whether human airway smooth muscle cells express MMP-12 mRNA *in-situ*, LCM was performed on sections of human bronchial biopsies to collect purified smooth muscle cell populations (Figure 1A). Quantitative real-time RT-PCR analysis revealed that these *in-situ *ASMC expressed MMP-12 mRNA (Figure 1B,C). The smooth muscle cells obtained from normal volunteers showed some expression of MMP-12, but there was elevated expression in the cells obtained from patients with asthma, COPD and chronic cough, however statistical significance was not achieved.

**Figure 2:**
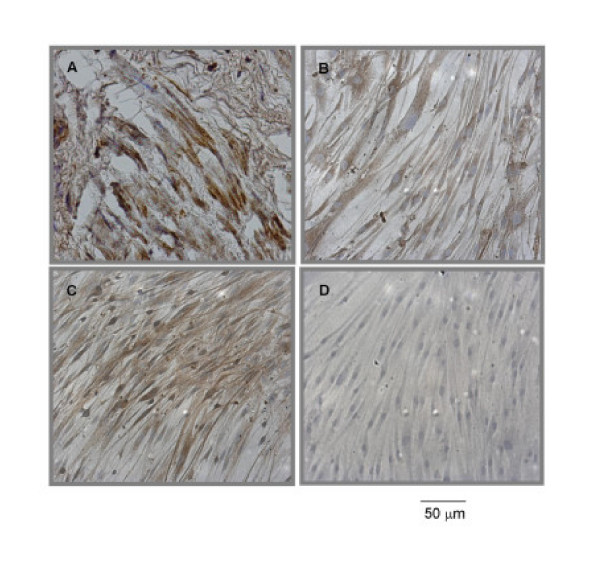
Expression of MMP-12 protein in bronchial smooth muscle tissue and cell cultures by immunostaining. Sections from human bronchial samples were prepared (A). Cell cultures were incubated on 8-well chamber slides for 72 hours in the absence (B) or presence (C) of 10 ng/ml IL-1β. Immunostaining was performed to detect MMP-12 expression using a rabbit anti-MMP-12 antibody. The primary antibody was replaced by a normal rabbit immunoglobulin as a negative control (D). Bar = 50 mm. Results are representative from three donors.

Immunohistochemistry of human bronchial samples showed positive immunostaining for MMP-12 in smooth muscle cells (Figure 2A), with no staining in the negative control section in which the rabbit anti-MMP-12 antibody was replaced by a normal rabbit immunoglobulin (data not shown).

### Stimulation of MMP-12 gene and protein expression by IL-1β in primary ASMC cultures

**Figure 3:**
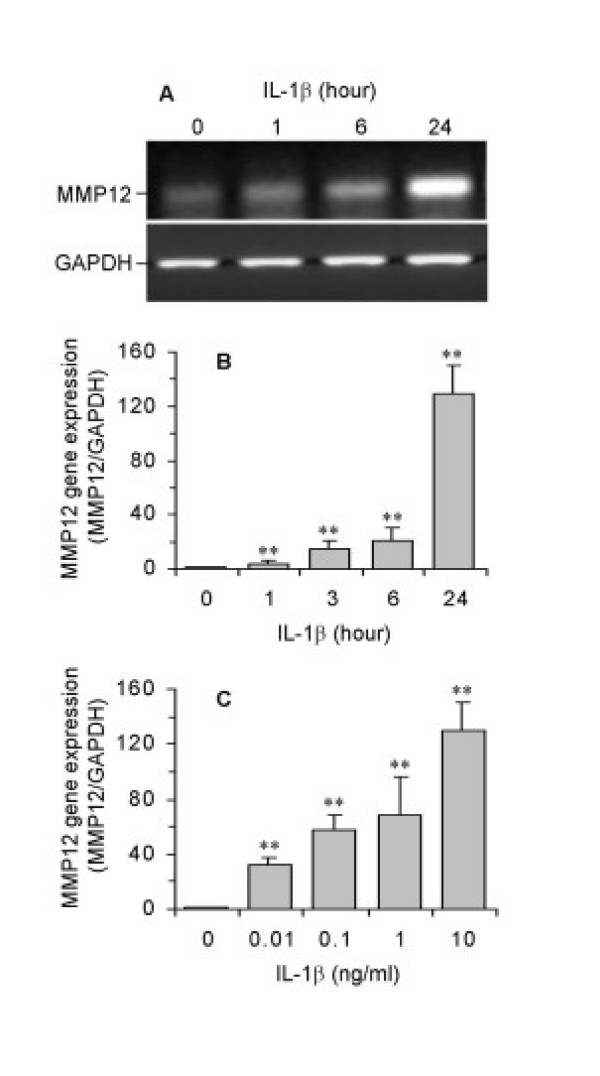
Stimulation of MMP-12 mRNA expression by IL-1β in ASMC. Cells were incubated in the absence or presence of IL-1β at 10 ng/ml for 1–24 hours (A, B), or for 24 hours over 0.01 to 10 ng/ml (C). MMP-12 and GAPDH mRNA expression was analysed by RT-PCR (A) or real-time RT-PCR (B,C). Results (B,C) were expressed as a ratio of target gene to GAPDH mRNA control and are the mean ± SEM from three ASMC donors. **P < 0.01 compared with control.

IL-1β (10 ng/ml) induced a time-dependent increase in MMP-12 mRNA expression analysed either by RT-PCR (Figure 3A) or quantitative real-time RT-PCR (Figure 3B). The increase was observed as early as 1 hour following treatment, with a maximal increase after 24 hours (Figure 3B). The effect of IL-1β was also concentration-dependent over the range of 0.01 to 10 ng/ml (Figure 3C). A 30-fold enhancement was observed at 0.01 ng/ml with a maximal effect of 130-fold at 10 ng/ml compared to unstimulated controls.

To determine whether IL-1β regulates MMP-12 protein production, ASMC cultured on chamber slides were treated with IL-1β (10 ng/ml) for 72 hours and MMP-12 expression in these cells was detected by immunocytochemistry. Cultured ASMC expressed MMP-12 protein in the absence (Figure. 2B) and presence (Figure. 2C) of IL-1β.

**Figure 4:**
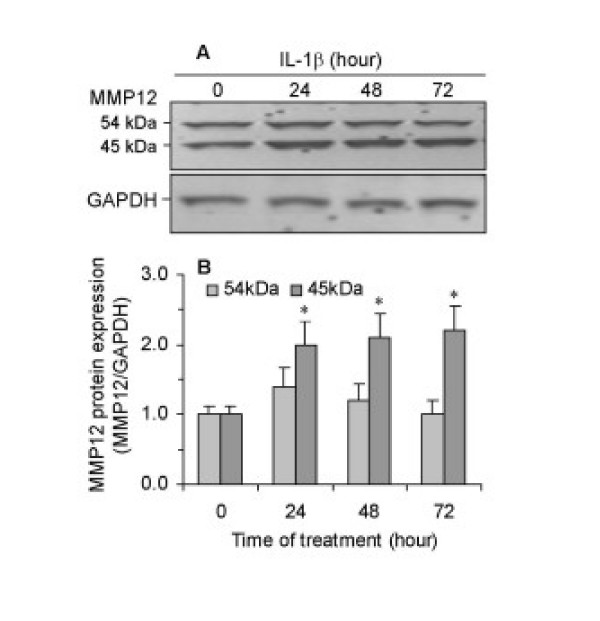
Western blot analysis of MMP-12 protein expression in ASMC and the effect of IL-1β. Cells were incubated in 6-well plates in the absence or presence of 10 ng/ml IL-1β for 24–72 hours. MMP-12 protein expression was analysed by Western blot (A) and shown as a ratio of GAPDH obtained by densitometric analysis (B). Results are mean ± SEM from three ASMC donors.

Western blot analysis using an anti-human MMP-12 antibody, that specifically recognises both the 54 kDa latent form and the 45 kDa active form of MMP-12, showed that unstimulated ASMC expressed both latent and active forms of MMP-12 (Figure 4). IL-1β caused a 2-fold increase in the expression of the active form after 24 hours, and this was sustained for up to 72 hours. There was no significant effect of IL-1β on the expression of the latent form of MMP-12.

### Secretion of MMP-12 protein by ASMC and enhancement by IL-1β

**Figure 5:**
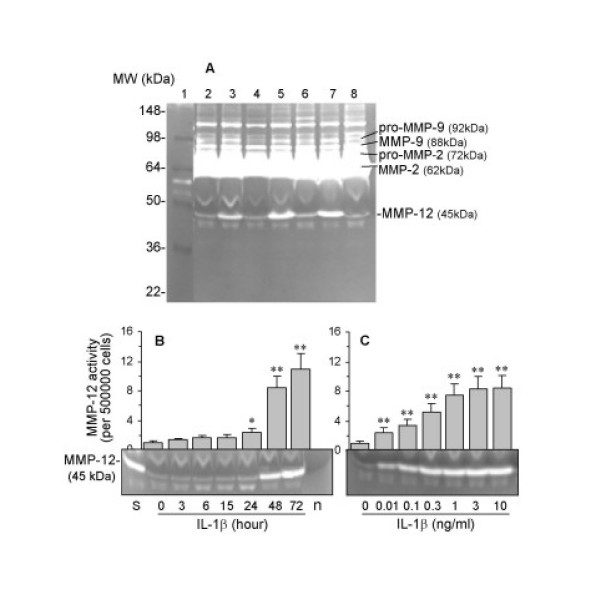
Activity of MMP-12 secreted by ASMC and stimulation by IL-1β. (A) Gelatin zymography detection of activity of MMPs released into the conditioned media by ASMC treated for 48 hours (lane 1, protein markers; lane 2, control; lane 3, 10 ng/ml IL-1β; lane 4, 10 ng/ml TGF-β; lane 5, TGF-β plus IL-1β; lane 6, 10 ng/ml TNF-α; lane 7, TNF-α plus IL-1β; lane 8, 10 ng/ml IL-13). (B) Time-dependent stimulation of MMP-12 activity by 10 ng/ml IL-1β. Lane s, human MMP-12 standard protein used as a positive control. Lane n, negative control. (C) Concentration-dependent stimulation of MMP-12 activity by IL-1β for 48 hours. Relevant band intensities were quantified by scanning densitometric analysis and normalized to 5 × 10^5 ^cells. Results are the mean ± SEM from three ASMC donors. *P < 0.05, **P < 0.01 compared with control.

To assess MMP-12 secretion and activity of ASMC, we performed gelatin zymography on conditioned media. As shown in Figure 5A, ASMC secreted several MMPs. In accordance with their molecular weights and with previous studies, these MMPs included 62 kDa MMP-2, 72 kDa pro-MMP-2, 88 kDa MMP-9 and 92 kDa pro-MMP-9, in descending order of magnitude of activity observed. In addition, a 45 kDa MMP-12 active form was observed; the molecular weight was confirmed by running an MMP-12 positive control protein (Figure 5B, lane S). Unstimulated ASMC secreted a detectable active form of MMP-12, with a time-dependent enhancement of the secretion after exposing cells with 10 ng/ml IL-1β (Figure 5B). A significant increase in MMP-12 activity was seen following 24 hour treatment, reaching a maximal ten-fold increase after 72 hours. Similar to the effects on MMP-12 mRNA, IL-1β also caused a concentration-dependent increase in the enzyme release from ASMC after 48 hours (Figure 5C): at 0.01 ng/ml, the activity increased by 2.5-fold, with a maximal effect at 10 ng/ml of an 8.5-fold increase. We also used casein zymography to detect MMP-12 activity and similar results were obtained albeit at a lower sensitivity compared to gelatin zymography (data not shown).

### Regulation of MMP-12 activity and gene expression by MAPKs, PI3-K and corticosteroid

**Figure 6:**
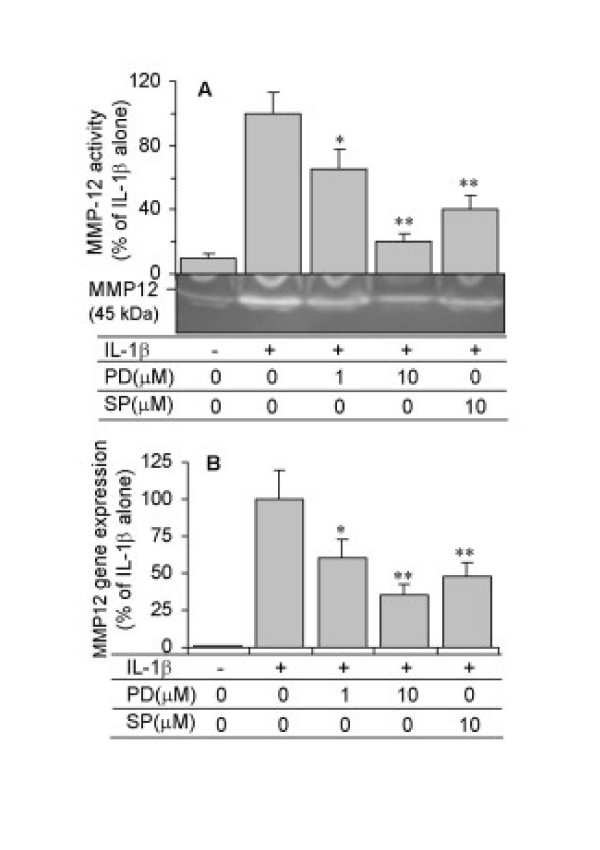
Inhibition of IL-1β-stimulated MMP-12 activity and mRNA expression by PD98059 and SP600125. ASMC were pre-treated for 1 hour with a specific inhibitor for ERK, PD98059 (1 or 10 μM) or for JNK, SP600125 (10 μM), and then were co-treated with 10 ng/ml IL-1β. (A) MMP-12 activity in conditioned media was detected after 48 hours by zymography. The relevant band intensities were quantified by scanning densitometric analysis. (B) MMP-12 mRNA was analysed after 24 hours by real-time RT-PCR. The data are expressed as the percentage of IL-1β alone and are the mean ± SEM from three ASMC donors. *P < 0.05, **P < 0.01 compared with IL-1β alone.

**Figure 7:**
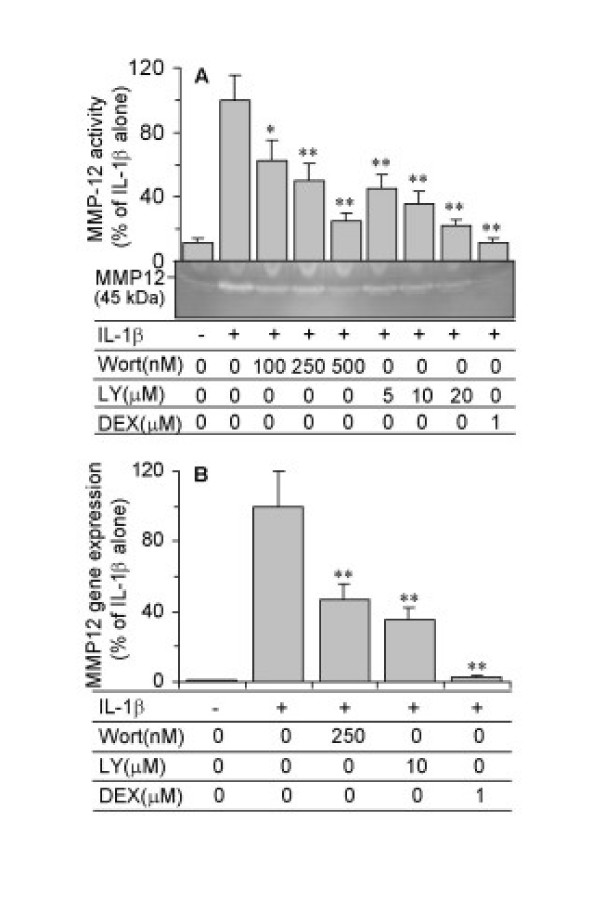
Inhibition of IL-1β-stimulated MMP-12 activity and mRNA expression by wortmannin, LY294002 and dexamethasone. ASMC were pre-treated for 1 hour with PI3-K inhibitors, wortmannin or LY294002, or dexamethasone at the indicated concentrations, and then were co-treated with 10 ng/ml IL-1β. (A) MMP-12 activity in conditioned media was detected after 48 hours by zymography. The relevant band intensities were quantified by scanning densitometric analysis. (B) MMP-12 mRNA was analysed after 24 hours by real-time RT-PCR. The data are expressed as the percentage of IL-1β alone and are the mean ± SEM from three ASMC donors. *P < 0.05, **P < 0.01 compared with IL-1β alone.

ASMC were pre-incubated for 1 hour with specific inhibitors for ERK (PD98059), JNK (SP600125), p38 MAPK (SB203580) or for PI3-K (wortmannin and LY294002), or with the corticosteroid, dexamethasone, and then co-treated with IL-1β(10 ng/ml) for 24 hours for analysis of mRNA expression or for 48 hours for protein activity. Treatment of ASMC with PD98059 (1–10 μM) or SP600125 (10 μM) inhibited IL-1β-induced MMP-12 activity (Figure 6A) and mRNA expression (Figure 6B). A significant inhibition in activity was seen with 1 μM PD98059 and 10 μM SP600125, with a >75% inhibition with 10 μM PD98059. SB203580 had no significant effect up to 1 μM (data not shown). Wortmannin (100–500 nM) and LY294002 (5–20 μM) induced a concentration-dependent inhibition of IL-1β-stimulated MMP-12 activity (Figure 7A). Dexamethasone (10^-6 ^M) also significantly blocked IL-1β-stimulated MMP-12 activity (Figure 7A). A similar suppression in IL-1β-induced MMP-12 mRNA expression was observed with wortmannin (250 nM), LY294002 (10 μM) and dexamethasone (10^-6 ^M) (Figure 7B).

### Effects of TNF-α, TGF-β1, IL-4 and IL-13 on IL-1β-induced MMP-12 release, gene expression and c-Jun activation

**Figure 8:**
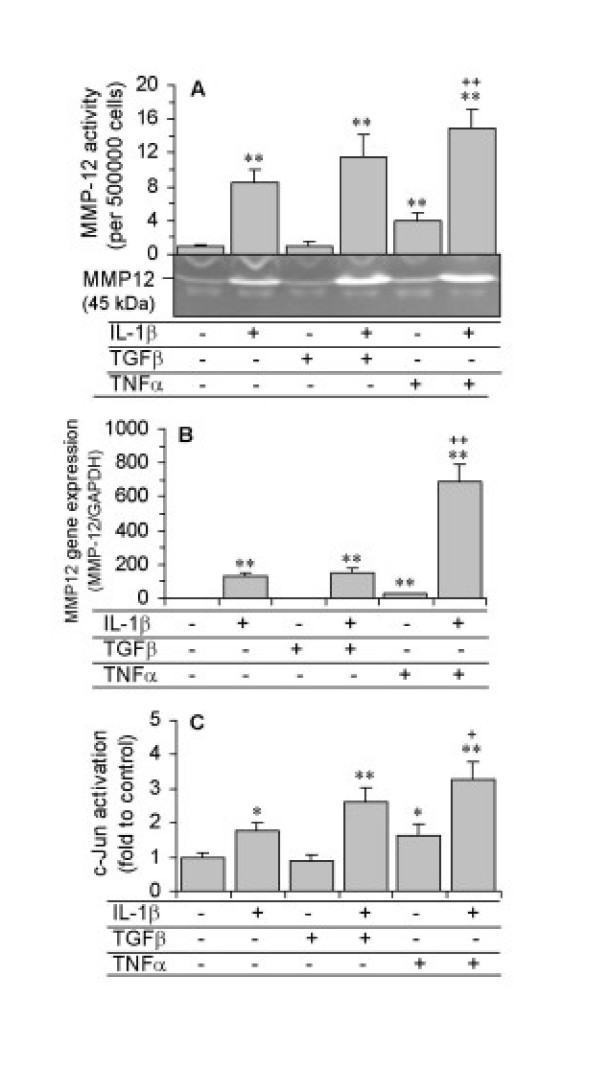
Effects of TNF-α and TGF-β1 on IL-1β-stimulated MMP-12 activity, mRNA expression and c-Jun activation. ASMC were treated with 10 ng/ml of TNF-α or TGF-β1 alone, or in combination with 10 ng/ml IL-1β. (A) MMP-12 activity in condition media was detected by zymography after 48 hours, and the relevant band intensities were quantified by densitometric analysis and normalized to 5 × 10^5 ^cells. (B) MMP-12 mRNA expression was determined by real-time RT-PCR after 24 hours and expressed as a ratio to GAPDH mRNA. (C) c-Jun activation and DNA-binding analysed by the TransAM AP-1 family kit. Data are the mean ± SEM from three ASMC donors. *P < 0.05, **P < 0.01 compared with control; +P < 0.05, ++P < 0.01, compared with IL-1β alone.

ASMC were incubated with 10 ng/ml of TNF-α, TGF-β1, IL-4 or IL-13 alone, or in combination with IL-1β (10 ng/ml) for 48 hours. TNF-α stimulated MMP-12 release by 4-fold in comparison with control levels, and also enhanced IL-1β-induced MMP-12 release. In contrast, TGF-β1 had no significant effect on basal or IL-1β-stimulated MMP-12 activity (Figure 8A). Similar results were observed for MMP-12 gene expression of ASMC after 24 hour treatment (Figure 8B). IL-1β-stimulated c-Jun activation and DNA binding was enhanced 85% by TNF-α (Figure 8C). TNF-α alone also had a significant effect on c-Jun activation and DNA binding. TGF-β1 had no significant effect on basal or IL-1β-induced c-Jun activation (Figure 8C). The T helper lymphocyte 2-derived (Th2) cytokines, IL-4 and IL-13, also had no effect on MMP-12 activity, gene expression or c-Jun activation either in the presence or absence of IL-1β (data not shown).

### Western blot analysis of MMP-12 secretion by airway smooth muscle cells

**Figure 9:**
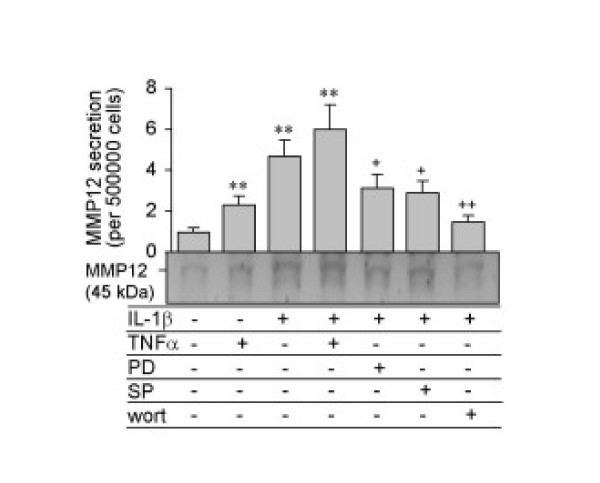
Western blot analysis of MMP-12 secretion by ASMC. Cells were incubated for 48 hours with 10 ng/ml IL-1β or TNF-α alone, or IL-1β combination with TNF-α, PD98059 (1 μM), SP600125 (10 μM) or wortmannin (250 nM) for 48 hours. MMP-12 protein released into conditioned media was determined by Western blot using 20 μl of 40-fold concentrated samples. Relevant band intensities were quantified by scanning densitometric analysis and normalized to 5 × 10^5 ^cells. Results are the mean ± SEM from three ASMC donors. **P < 0.01 compared with control; +P < 0.05, +P < 0.01 compared with IL-1β alone.

We also performed Western blot analysis to determine MMP-12 secretion by using a specific MMP-12 antibody (Figure 9). Conditioned media were collected from 6-well plates after ASMC were treated for 48 hours with IL-1β and/or TNF-α in the absence or presence of the specific inhibitors. The results of MMP-12 secretion analysed by Western blot was consistent with the data obtained by gelatin zymography and confirmed that ASMC secreted the 45 kDa active form of MMP-12 which was up-regulated by inflammatory cytokines, IL-1β and TNF-α. The specific inhibitors for ERK, JNK and PI3-K also down-regulated MMP-12 secretion by IL-1β.

## Discussion

We performed laser capture microdissection to collect smooth muscle cells from bronchial biopsy sections and found that *in-situ *ASMC expressed both MMP-12 mRNA and protein. MMP-12 mRNA expression was found in ASMC obtained from normal subjects, and was somewhat higher in patients with asthma, COPD and chronic idiopathic cough. More patients are likely to be needed to demonstrate statistical significance. In cultured primary ASMC, we have also shown that MMP-12 mRNA and protein expression and secretion were regulated by the inflammatory cytokine, IL-1β. IL-1β induced a >100-fold increase in the mRNA levels and a >10-fold enhancement in the enzyme release and activation from ASMC cultures, that was mediated by mechanisms involving ERK, JNK, PI3-K and AP-1 pathways. Therefore, airway smooth muscle cells, similar to vascular smooth muscle cells, are an important source of MMP-12 [23].

Potential regulators of MMP-12 in the inflammatory milieu of the airways include inflammatory cytokines and growth factors. The inflammatory cytokines, IL-1β and TNF-α, and the growth factor, TGF-β1, are thought to play active roles in asthma and emphysema/COPD [24-26], and induce the induction of a number of inflammatory mediators by ASMC [27,28]. Increased expression of IL-1β has been detected in airway epithelial cells and alveolar macrophages of patients with asthma [29,30]. The expression of TNF-α and TGF-β1 are elevated in lung and bronchoalveolar lavage fluid in asthma [31,32]. We found that IL-1β induced a time- and concentration-dependent increase in MMP-12 mRNA expression. Although the maximal stimulation of MMP-12 mRNA expression by IL-1β reached 130-fold of control levels, the protein expression of the 45 kDa active form was only increased by 2-fold, with a slightly increase in the 54 kDa latent form, likely to be due to its conversion to the 45 kDa active form. Since the pathological significance of MMP expression depends on its secretion and activity and since most cells synthesize and immediately secrete MMPs into the extracellular environment [33], we next examined whether IL-1β enhanced MMP-12 release from ASMC. Indeed, IL-1β enhanced MMP-12 secretion and activity of ASMC in a time- and concentration-dependent manner, with an up to 10-fold increase to control levels, as determined by gelatin zymography. Our data are similar to previous studies on MMP-12 release by macrophages [17,18]. The difference in levels of mRNA and protein suggest a complex regulation of MMP-12 translation and secretion. This allows control to be exerted at distinct levels preventing excessive release of MMP-12 unless further stimulatory signals are received.

We also found that TNF-α stimulated MMP-12 gene expression and activity of ASMC although to lesser extent than IL-1β, as has been described in chondrocytes [19]. TNF-α also had an additive effect with IL-1β in MMP-12 activity, although in terms of MMP-12 mRNA expression, this was synergistic. TGF-β1 had no significant effect on MMP-12 activity and gene expression, which is in contrast to the report of TGF-β1 inhibition of IL-1β-mediated MMP-12 induction in macrophages [18]. This suggests differential effects of TGF-β1 on MMP-12 regulation in different cell types. We did not observe regulation of MMP-12 mRNA levels and enzyme secretion when ASMC were exposed to the Th2 cytokines IL-4 or IL-13, either alone or in combination with IL-1β, although these cytokines can induce MMPs in mouse lung tissue [34]. MMP-12 induction in human bronchial epithelial cells by TNF-α, epidermal growth factor and interferon-γ but not by IL-4 or IL-13 has recently been reported [35]. Overall, these data imply that MMP-12 release from ASMC is under the control of select pro-inflammatory stimuli and is regulated differently between human and murine cells.

AP-1 is a dimeric complex composed of Jun (c-Jun, JunB or JunD) and Fos (FosB, c-Fos, Fra-1 or Fra-2) proteins, which may be involved in the modulation of MMP-12 as has been shown in macrophages [18] and vascular smooth muscle cells [23]. Removal of the AP-1 binding site from the MMP-12 promoter abolished the basal and inducible expression of MMP-12 [23]. c-Jun, which is a predominant component of the AP-1 binding complex binding to the MMP-12 promoter [23], can potentially transactivate the MMP-12 promoter up to 20-fold in macrophages [18]. Therefore, we examined whether these cytokines affected MMP-12 secretion mediated through regulation of c-Jun activity in ASMC. We found that IL-1β and TNF-α enhanced c-Jun activation and nuclear binding, and when combined together, they had an additive effect. TGF-β1 alone had no effect, and barely augmented IL-1β-induced c-Jun activation. The effects of these cytokines on c-Jun activation were directly correlated with their activities on MMP-12 release. This combination with the effect of JNK inhibitor implies a role for c-Jun in mediating cytokine-stimulated MMP-12 induction in ASMC.

The intracellular mechanisms and signaling pathways that mediate IL-1β-induced MMP-12 in ASMC are unknown. IL-1β stimulates the induction of MMP-1 in human gingival fibroblasts by activation of MAPKs [36]. MAPKs are a family of serine/threonine kinases, and at least three subfamilies that differ in their substrate specificity have been characterized: ERK, JNK and P38 MAPK. Here, we show that ERK and JNK, but not p38 MAPK, pathways are involved in IL-1β-induced MMP-12 secretion and gene expression. IL-1β-induced MMP-3 and -13 gene and protein expression in articular chondrocytes and MMP-9 expression in rat brain astrocytes have also been reported to be regulated by ERK and JNK pathways [37,38]. We have previously shown that at a concentration of 10 μM, SP600125 induces specific inhibition of IL-1β-induced JNK activation in ASMC, having no effect on p38 MAPK and ERK activation [39]. We did not use concentrations of SB20358 higher than that of 1 μM, since above this concentration this compound inhibits the JNK pathway [40]. Therefore, our data indicate that the induction of MMP-12 by IL-1β in ASMC may not involve the participation of the p38 MAPK pathway, which is contrary to the regulation of MMP-3, -9 and -13 in articular chondrocytes and rat brain astrocytes [37,38]. These differences may reflect different cell types and MMPs studied.

PI3-kinase is involved in the regulation of a number of cellular responses, including MMP-12 induction in human vascular smooth muscle cells [23]. We used two structurally different inhibitors of PI3-K: wortmannin, a non-reversible inhibitor which covalently binds to the catalytic subunit of PI3-K [41], and LY294002, a reversible inhibitor that competes with ATP for the PI3-K substrate-binding site [42]. Our results indicate that PI3-K is required for IL-1β-stimulated MMP-12 mRNA expression and secretion in ASMC. In vascular smooth muscle cells, PI3-kinase activation appears to be required for MMP-12 transcriptional activity through AP-1 binding to the gene promoter [23].

Corticosteroids are anti-inflammatory drugs used for the treatment of asthma and COPD, and previous studies have shown their inhibitory effects on MMP-12 induction by lipopolysaccharide in human alveolar macrophages [7]. We observed marked down-regulation of IL-1β-stimulated MMP-12 mRNA expression and enzyme activity by dexamethasone. This indicates that corticosteroid treatment may lead to prevention of airway wall remodelling and the development of MMP-12-dependent emphysema in COPD although evidence for this in the airways of asthmatic and COPD patients is limited. It is not known, whether MMP-12 release from airway smooth muscle cells in COPD may be similarly inhibited by corticosteroids, since there is relative corticosteroid resistance in COPD.

## Conclusion

We have provided evidence that *in vivo *ASMC express MMP-12 mRNA and protein. The pro-inflammatory cytokine IL-1β stimulates a significant enhancement in MMP-12 gene expression, protein production and enzyme secretion, which is mediated by mechanisms involving ERK, JNK, PI3-K and AP-1 signaling pathways. Induction of MMP-12 by IL-1β is up-regulated by the inflammatory cytokine TNF-α and down-regulated by the corticosteroid dexamethasone. Exposure to the inflammatory cytokines, IL-1β and TNF-α, stimulates the release of MMP-12 which in turn activates other MMPs [10] to breakdown extracellular matrix proteins and promote inflammatory cells migration, and induces enhanced elastolytic activity and excessive airway remodeling. Thus, MMP-12 induction by inflammatory cytokines may be a potential pathophysiological mechanism by which ASMC mediate and facilitate inflammatory respiratory disorders such as asthma and COPD.

## Abbreviations

AP-1, activator protein-1

ASMC, airway smooth muscle cells

COPD, chronic obstructive pulmonary disease

ECM, extracellular matrix

ERK, extracellular signal-regulated kinases

GAPDH, glyceraldehyde-3-phosphate dehydrogenase

JNK, c-Jun N-terminal kinases

LCM, laser capture microdissection

IL, interleukin

MAPK, mitogen-activated protein kinase

MMP, matrix metalloproteinase

PCR, polymerase chain reaction

p-c-Jun, phosphorylated c-Jun

PI3-K, phosphatidylinositol 3-kinase

RT, reverse transcription

SM, smooth muscle

TGF, transforming growth factor

Th2, T helper lymphocyte 2-derived

TNF, tumour necrosis factor

## Competing interests

The author(s) declare that they have no competing interests.

## Authors' contributions

**SX **participated in the design and coordination, performed the experiments and data analyses and drafted the manuscript. **RI**, **MBS **and **UO **participated in the primary ASMC cultures. **PB **prepared reagents and participated in analysis of the data for real-time PCR of LCM samples. **AP **and **GC **carried out the immunostaining and prepared histological samples for immunohistochemistry. **IA **participated in coordinating the use of LCM system. **KFC **conceived the idea, participated in the design and coordination of the study, and wrote the manuscript. All authors have read and approved the final manuscript.
